# The Effect of Central Loops in miRNA:MRE Duplexes on the Efficiency of miRNA-Mediated Gene Regulation

**DOI:** 10.1371/journal.pone.0001719

**Published:** 2008-03-05

**Authors:** Wenbin Ye, Qing Lv, Chung-Kwun Amy Wong, Sean Hu, Chao Fu, Zhong Hua, Guoping Cai, Guoxi Li, Burton B. Yang, Yaou Zhang

**Affiliations:** 1 Department of Biological Science and Biotechnology, Tsinghua University, Beijing, People's Republic of China; 2 Division of Life Science, Graduate School at Shenzhen, Tsinghua University, Shenzhen, People's Republic of China; 3 Sunnybrook Research Institute, Sunnybrook Health Sciences Centre, Department of Laboratory Medicine and Pathology, University of Toronto, Toronto, Canada; 4 Shenzhen Beike Biotechnology Co., Ltd., Shenzhen, People's Republic of China; Center for Genomic Regulation, Spain

## Abstract

MicroRNAs (miRNAs) guide posttranscriptional repression of mRNAs. Hundreds of miRNAs have been identified but the target identification of mammalian mRNAs is still a difficult task due to a poor understanding of the interaction between miRNAs and the miRNA recognizing element (MRE). In recent research, the importance of the 5′ end of the miRNA:MRE duplex has been emphasized and the effect of the tail region addressed, but the role of the central loop has largely remained unexplored. Here we examined the effect of the loop region in miRNA:MRE duplexes and found that the location of the central loop is one of the important factors affecting the efficiency of gene regulation mediated by miRNAs. It was further determined that the addition of a loop score combining both location and size as a new criterion for predicting MREs and their cognate miRNAs significantly decreased the false positive rates and increased the specificity of MRE prediction.

## Introduction

MicroRNAs (miRNAs) are small non-coding RNAs which guide translational repression[1]–[4] or RNA cleavage[5], [6] by binding partially or with perfect complementarity with their target sites in the 3′-untranslated region (3′-UTR) of mRNAs. Having been studied for about a decade, miRNAs have been demonstrated to have important roles in development[7], [8], cell proliferation [9]–[11], differentiation [12]–[16], apoptosis[17], [18], cell cycle progression [19]–[21], tumorigenesis[22]–[26] and many other physiological or pathological processes[2], [16], [27]. Hundreds of miRNAs have been identified by experimental or bioinformatics methods in animals, plants, and viruses, and the number of miRNAs are still increasing[28]. However, the comprehension of miRNA function remains preliminary, because few miRNA targets have been identified in spite of the enormous quantity of identified miRNAs[5], [29], [30]. It therefore appears that progress in our understanding of miRNA function has been limited by the difficulty in accurate target prediction and validation.

Identification of miRNA targets or the miRNA recognizing element (MRE) is the first step towards understanding the biological function of miRNAs. Since experiments used for the identification of miRNA targets are laborious and are hard to apply for large-scale investigations, many computational approaches have been recently developed to predict miRNA targets in vertebrates and *Drosophila* [31]–[38]. However, computational algorithms predicting miRNA targets still have high false-positive rates, because of the poor understanding of the interaction between miRNAs and their cognate mRNAs. Generally, miRNA:MRE duplexes consist of a 5′ end seed region, central bulges or loops (loop region or mismatches), and a 3′ end tail region. The importance of the seed region in miRNA-mediated gene regulation is highly emphasized by most researchers, while the effect of the tail region has been addressed computationally by Enright *et al.* and John *et al.*, and also experimentally by Vella *et al.*, as well as others [34], [37], [39]. However, the role of the loops in the middle region of the miRNA:MRE duplex is not very clear, though Kiriakidou and coworkers have reported that the size of the loop or bulge might affect the efficiency of miRNA-mediated gene regulation[32].

In this study, we used vascular endothelial growth factor (VEGF) as a model to examine the effect of the loop region in miRNA:MRE duplexes on miRNA-mediated gene regulation, and found that the location of the central loop is another one of the important factors in miRNA functioning. VEGF is an ideal model because the numbers of miRNA binding sites and the various types of miRNA:MRE structures were predicted in the VEGF 3′-UTR in our previous investigation[40]. The addition of loop scores combining the effects of both location and size as a new criterion for the prediction of MREs and their cognate miRNAs to *FindTar,* a computational algorithm developed in our lab, decreased the false positive rates and increased the accuracy of MRE prediction significantly.

## Results

### Putative miRNAs for VEGF regulation

**Figure 1:**
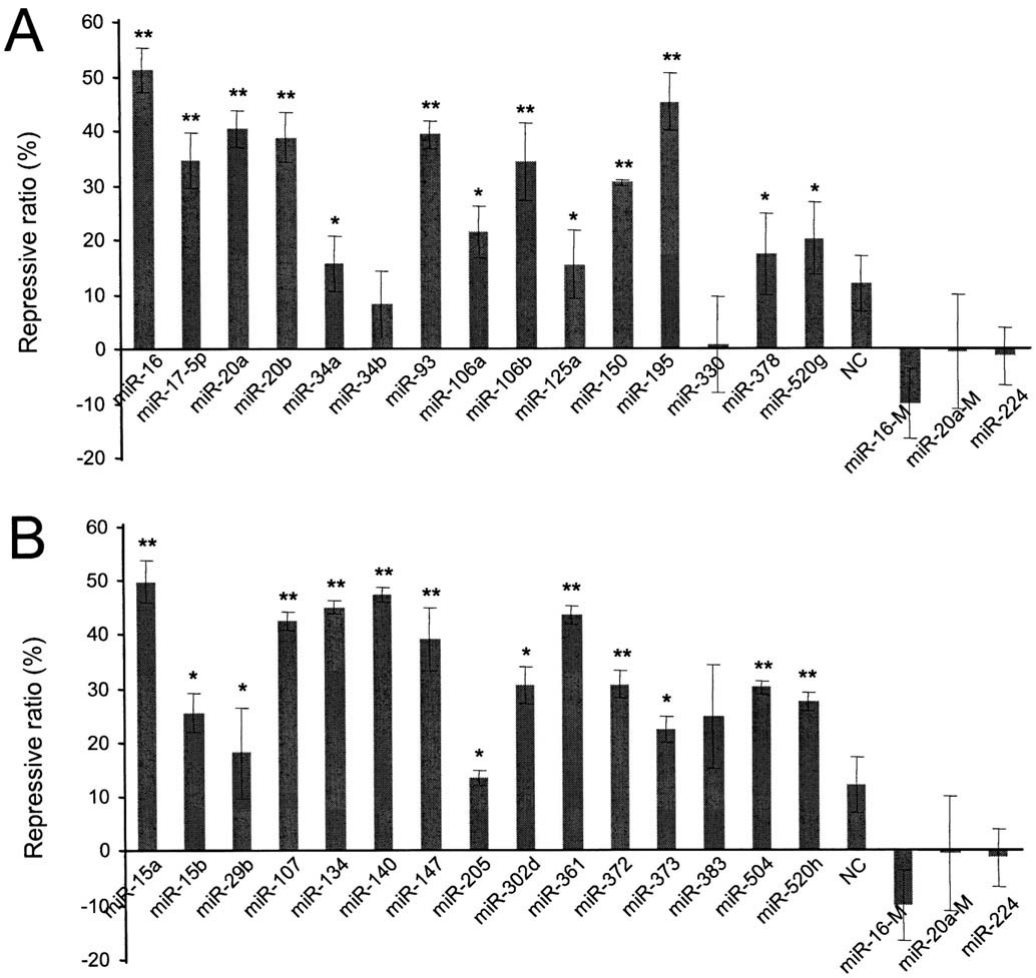
Validation of VEGF regulation by putative miRNAs. The effect of putative VEGF-regulative miRNAs on VEGF expression was tested in hypoxia-induced CNE cells by transfecting the cells with siRNA duplexes homologous in sequence to the miRNAs in group 1 (A) and 2 (B). VEGF expression was determined by ELISA. MiRNAs in group 1 were selected using a strict criteria: free energy less than −24 kcal/mol, nt2–7 perfectly pairing with the 5′-end of miRNAs, and sequence conservation of target sites across five vertebrate species. MiRNAs in group 2 were chosen from miRNAs that only met relatively relaxed criteria: free energy between −24 kcal/mol and −15 kcal/mol, and an elastic seed window tolerating one G:U wobble in successive 6-mers. Repressive ratio = (1–ELISA value of miRNA/ELISA value of blank)×100%. The blank is the sample from cells without transfection, providing a control for protein expression in the absence of regulation. A random sequence (NC), *miR-224*, mutated *miR-16* (*miR-16-M*), and *miR-20a* (*miR-20a-M*) that do not have MREs in the VEGF 3′-UTR were used as negative controls.

VEGF is an important regulator of physiological or pathological angiogenesis and is involved in embryogenesis, wound healing, and tumorigenesis [41]. In this study, we used VEGF as a model to investigate the effect of the secondary structure of the non-seed region in the miRNA:MRE duplex on miRNA-mediated gene regulation. Previously, we developed an algorithm, *FindTar* version 1.0, using the generally recognized criteria to predict the binding interactions of miRNA:MREs and their secondary structures. The cut-offs −24 and −15 kcal/mol were chosen subjectively to avoid predicting too few or too many putative miRNAs targeting VEGF. When strict screening criteria were used, which included a minimal free energy (ΔG) below −24 kcal/mol, tail scores of miRNA:MREs duplexes above 15, seed windows at nt2–7 with conservation without G-U wobble, 16 MREs for 15 potential miRNAs were predicted as Group 1 (see Table S1A) in the VEGF 3′-UTR. When we relaxed the screening criteria to ΔG between −24 and −15 kcal/mol, tail score above 5, plus elastic seed windows (nt1–6, nt2–7, or nt3–8) with tolerance for a single G-U wobble in the seed region[42], hundreds of MREs and miRNAs were predicted. From these miRNAs, we randomly chose 15 miRNAs as the experimental Group 2 (Table S1B). Hypoxia-induced CNE cells (nasopharyngeal carcinoma cell line) were transfected with synthetic miRNA duplexes, including all the miRNAs in Group 1 and 2. A random sequence (NC), m*iR-224*, mutated *miR-16*, and *miR-20a* that did not have MREs in the VEGF 3′-UTR were used as negative controls. VEGF expression was measured by ELISA. The experiments indicated that 23 of the 30 miRNAs significantly repressed VEGF expression by over 20%, compared to negative controls (Figure 1).

**Figure 2:**
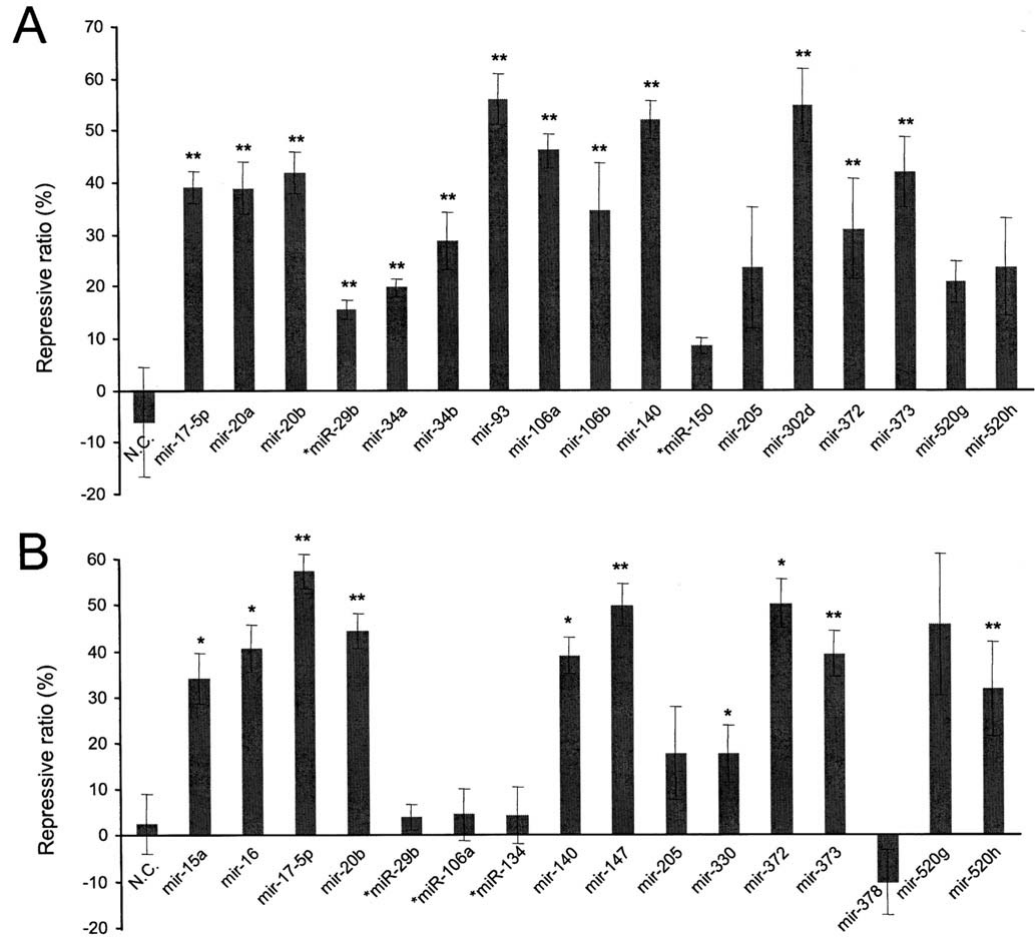
Luciferase activity assay. COS-7 cells were co-transfected with the luciferase reporter vector, which contained either the VEGF 3′-UTR fragment nt31-216 (pRL-VEGF-Con1) or nt703-944 (pRL-VEGF-Con2), and an miRNA which has a putative binding site in either pRL-VEGF-Con1 (A) or pRL-VEGF-Con2 (B). Luciferase activity was measured to determine the effect of these miRNAs on luciferase translation. All miRNAs have only one predicted MRE in the corresponding report vector. Repressive ratio = (1–LA value of miRNA/LA value of blank)×100%. LA: luciferase activity. The blank is the sample from cells without transfection, providing a control for protein expression in the absence of regulation. A random sequence (NC), *miR-29b*, *miR-150*, *miR-106b*, and *miR-134* that do not have MREs in the VEGF 3′-UTR were used as negative controls.

To confirm the repressive action of the miRNAs through forming miRNA:MRE duplexes, we inserted two fragments of the VEGF 3′-UTR into a luciferase expression vector, generating the luciferase reporter constructs pRL-VEGF-Con1 and pRL-VEGF-Con2. pRL-VEGF-Con1 contains a fragment located at nt31-216 of the VEGF 3′-UTR, while pRL-VEGF-Con2 contains a fragment at nt703-944. Of the 30 miRNAs tested in the ELISA assay, 21 harbored a single MRE in pRL-VEGF-Con1 and/or pRL-VEGF-Con2. These miRNAs were co-transfected with their respective reporters into COS-7 cells and the levels of *Renilla* luciferase (RL) were measured to determine the repressive effects of these miRNAs. These experiments indicated that most of the selected miRNAs produced a direct effect with significant repression of luciferase activity as compared with the controls (Figure 2).

### The relationship between the central loops of miRNA:MRE duplexes and the repressive efficiency of miRNAs

**Figure 3:**
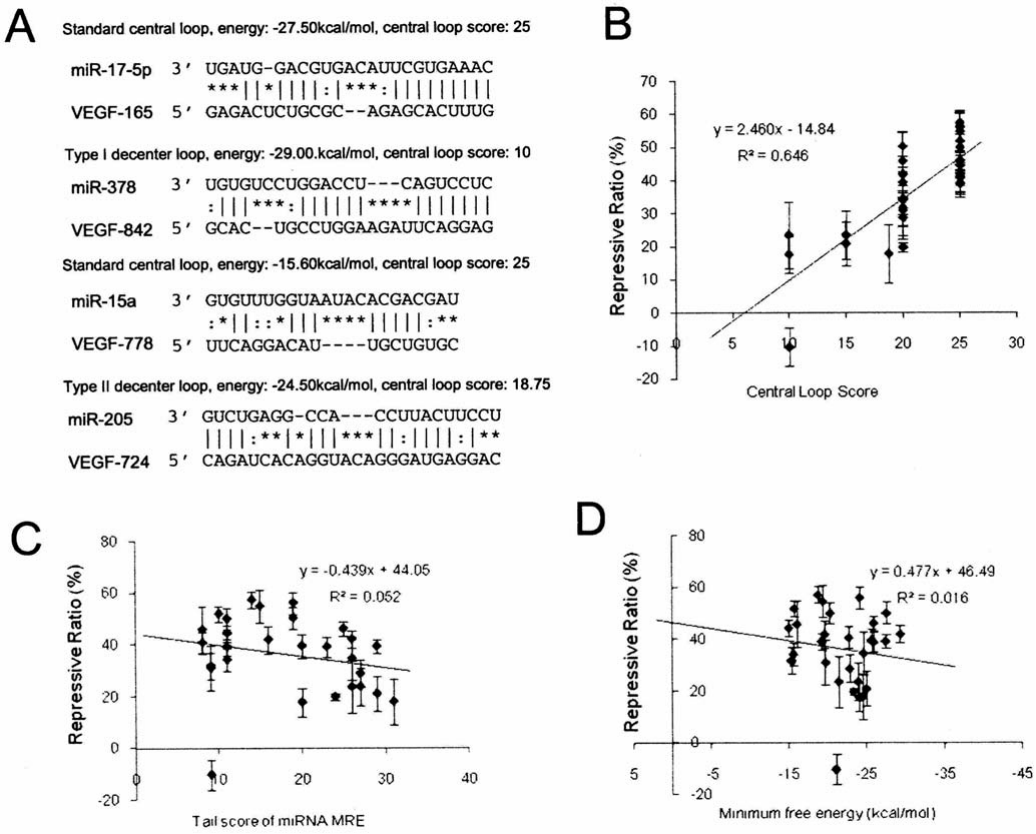
Correlation and linear regression analysis of central loops of miRNA:MRE duplexes. A, Central loops (or bulges) of miRNA:MRE duplexes were divided into three categories: standard loop, type I decentered loop, and type II decentered loop. Standard loops start at between nt9 and nt11 of the miRNA:MRE duplex counting from the 5′ end of the miRNAs. The type I decentered loop starts before nt9, with the type II decentered loop opening after nt11. The loop score is designated according to the algorithm introduced in the Methods. B–D, Relationship between miRNA repression efficiency and the central loop score (B), tail score (C), and minimum free energy (D). Compared with the tail score and minimum free energy, the central loop score has a closer correlative relationship with miRNA repression efficiency (r = 0.646, p<0.001).

We analyzed the relationship between the central loop of miRNA-mRNA duplexes and the repressive efficiency of miRNAs in Groups 1 and 2 on VEGF expression, and found that the location of the central loop in miRNA:MRE duplexes appeared to play an important role. The loops of the miRNA:MRE duplexes in Table S1 were divided into three types according to their location in the duplex. Loops starting between nt9 and nt11 inclusive of the miRNAs were designated standard loops, before nt9 as Type I decentered loops, and after nt11 as Type II decentered loops (Figure 3A). Thus, of the 30 total miRNA:MREs tested, there were 21 miRNAs with standard central loops, 2 with Type I decentered loops, and 7 with Type II decentered loops. Of the 24 functional miRNAs with a repression ratio of VEGF expression >20%, 19 harbored a standard central loop, and only 5 contained Type II decentered loops. On the other hand, amongst the 7 non-functional miRNAs, 2 had Type II decentered loops, 2 had Type I decentered loops, and only 3 had standard central loops. Our analysis indicates that most of the MREs with a decentered loop were either false positives or highly inefficient. According to these data, we hypothesize that the central loop is one of the important factors that guide miRNA-MRE interactions. However, our data only included interactions between miRNAs and the 3′-UTR of VEGF, and many VEGF regulatory miRNAs have more than one MRE in the 3′-UTR of VEGF with an overlap of target sites, and there is a degree of similarity between different microRNAs to each other. Therefore, more data was needed to confirm our hypothesis on the importance of the loop in the miRNA:MRE duplexes in miRNA functioning.

The published data on miRNA target identification by experiments and bioinformatics approaches, which were available on the website DIANA Tarbase (www.diana.pcbi.upenn.edu/tarbase.html)[31], [32], [43]–[49], support our hypothesis. We used these data to validate the effect of the loop structure of miRNA:MRE duplexes on the repression of gene expression. To avoid the incongruency brought by different standards or outcomes of RNA secondary structure and free energy predicted by distinct algorithms, we re-calculated the secondary structures of miRNA:MRE duplexes and their free energy for all the predicted miRNAs obtain from the online database by RNAcofold, a program for the prediction of hybrid structures of two RNA sequences (www.tbi.univie.ac.at/ivo/RNA/)[50]–[52]. Thus, we provided a relatively equal platform or prerequisite to compare the structure of all these microRNA targets. To exclude the interference of multiple MREs of a single miRNA for one gene, we only took those miRNAs that have single MREs on one gene into account, and a total of 20 miRNAs were selected (Table S2). Analysis of the effect of the loop structure of miRNA:MRE duplexes on the repression of gene expression showed that 18 of the 20 microRNAs had their loop starting from p9 to p11, while only 2 had Type I decentered loops. Therefore, while much attention has focused on the seed region in the identification of miRNA targets[53], [54], it seems that other factors such as the location of the loops also have a great impact on gene repression.

The miRNAs with a single MRE in pRL-VEGF-Con1 and/or pRL-VEGF-Con2 (Table S3) were selected to investigate the relationship between miRNA repression efficiency and the secondary structure of miRNA:MRE duplexes. The central loops are scored as follows: (i) to get a location coefficient according to the locations of loops: 1 for standard loop, 0.75 for type II decentered loop, 0 or 0.5 for type I decentered loops starting before nt7 inclusive or at nt8; (ii) to get the size coefficient according to the sizes of the loops: 10 for 1 bp, 20 for 2 bp, and 25 for 3 bp. If the size of the loop was more then 3 bp, the size coefficient would drop to 20. The total loop scores were calculated by multiplying the location coefficient with the size coefficient. The relationship between miRNA efficiency and the central loop was analyzed using correlation and linear regression. The central loop scores demonstrated a significant correlative relationship with miRNA repressive efficiency (Figure. 3B). The tail score (Figure 3C) and minimum free energy (Figure 3D) did not show significant relationships with gene repression.

### Effect of loop location on miRNA action

**Figure 4:**
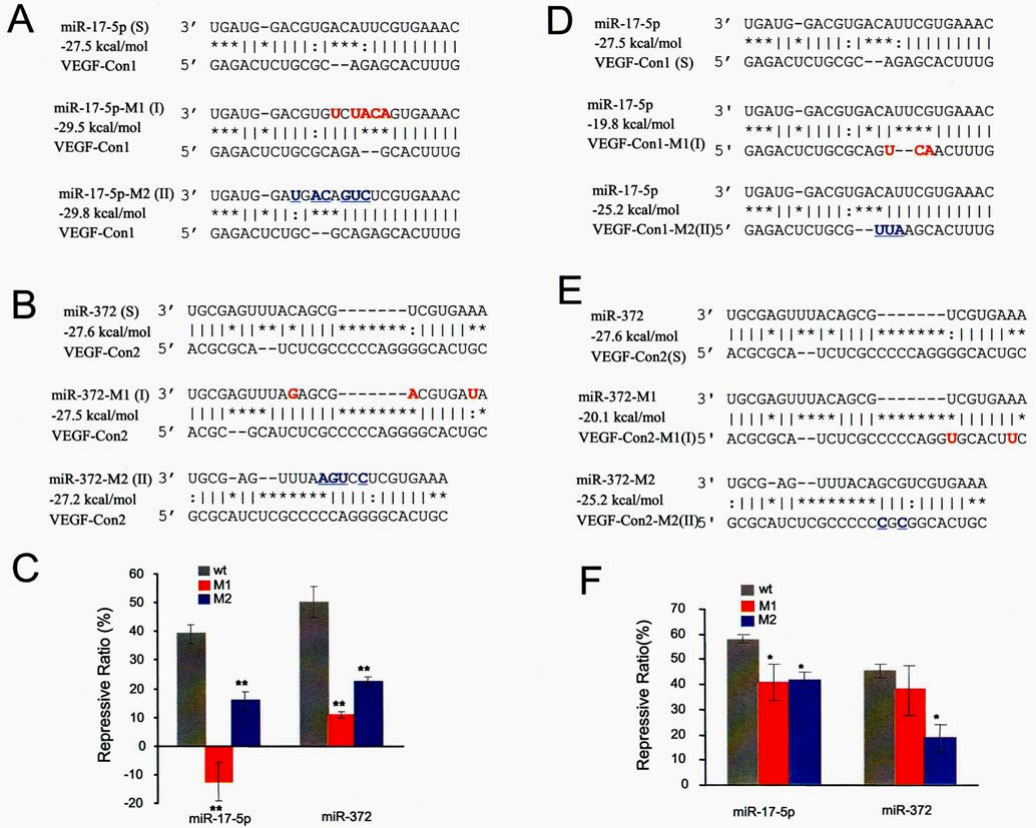
Mutation assays. Site-directed mutagenesis of *miR-17-5p* (A) , *miR-372* (B), pRL-VEGF-Con1 (D), and pRL-VEGF-Con2 (E) caused movement of the standard loops of miRNA:MRE duplexes, forming Type I and Type II decentered loops instead, while the free energy of miRNA:MREs was kept at similar levels. COS-7 cells were transfected with wild type or mutated miRNAs and different report vectors. The levels of luciferase activity decreased significantly due to the change in loop location (C and F).

Although the results of co-transfection of miRNAs and the reporter pRL-VEGF-Con1 and/or pRL-VEGF-Con2 suggested a tight connection between the loop structure and the repressive effect of miRNAs, direct evidence was still needed to confirm our hypothesis. To do so, we performed site mutation assays (Figure 4). Mutations of *miR-17-5p* (Figure 4A) and *miR-372* (Figure 4B) moved the central loops of the miRNA:MRE duplexes to Type I and Type II decentered loops while keeping the minimum free energy of the miRNA:MREs at similar levels, and the mutation of MREs of *miR-17-5p* and *miR-372* in pRL-VEGF-Con1 and pRL-VEGF-Con2 also formed Type I and Type II decentered loops (Figure 4D and E). COS-7 cells were then transfected with wild type or mutated miRNAs with different report vectors. The levels of luciferase activity were measured to determine the repressive effects of different miRNA:MRE duplexes. Duplexes with type I or II decentered loops produced significantly lower repressive effects, compared with the miRNA:MRE duplexes with standard loops (Figure 4C and F).

**Figure 5:**
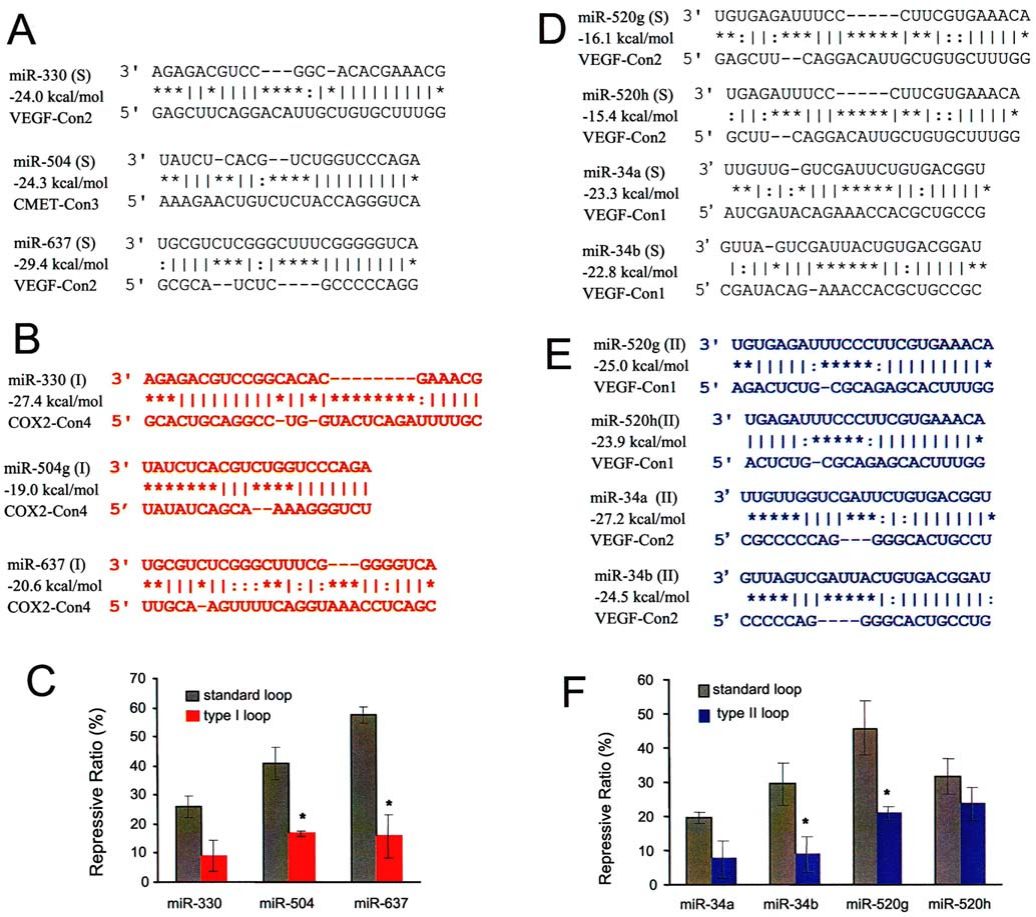
Effect of loop location on miRNA functioning. The insert fragments of the VEGF 3′-UTR in pRL-VEGF-Con1 and/or pRL-VEGF-Con2 were changed to fragments of the c-Met 3′-UTR or COX-2 3′-UTR to create pRL-CMET-Con3 and pRL-COX2-Con4. These changes allowed us to compare miRNA:MRE duplexes bearing standard central loops (A and D) in miRNA:MRE duplexes with the ones bearing Type I (B) and Type II (E) decentered loops. Luciferase activity assays indicated that changes to Type I and Type II decentered loops decreased the repressive effect of miRNAs significantly (C and F).

We then investigated the effect of loop location on miRNA functioning using naturally existing sites (Figure 5). We changed the fragments of the VEGF 3′-UTR in pRL-VEGF-Con1 or 2 to fragments of c-MET or COX2 3′-UTR, resulting in the constructs pRL-CMET-Con3 and pRL-COX2-Con4. Change of the 3′-UTR fragments allowed us to compare miRNA:MRE duplexes bearing standard central loops (Figure 5A and D) with those bearing Type I (Figure 5B) or Type II (Figure 5E) decentered loops. COS-7 cell were co-transfected with miRNAs and different report constructs, and the levels of luciferase activity measured. The experiments indicated that miRNA:MRE duplexes with Type I or Type II decentered loops caused by changing the fragments showed significantly lower repressive effects, compared with the miRNA:MRE duplexes with standard loops (Figure 5C and F). The results confirmed the importance of the location of central loops in miRNA:MRE in the repressive activity of miRNAs.

### The effect of adding loop scores as a screening criterion on MRE prediction

Since the central loop of miRNA:MRE appeared to be important for miRNA-mediated gene regulation, addition of a loop score as a criterion for MRE prediction should improve the results. To confirm this, all the single MREs in this investigation and MREs in the results (Figure 1, 3, and 6) of Kiriakidou's publication[32] were included to re-calculate the secondary structures of the miRNA:MRE duplexes by RNAcofold and screened by *FindTar* version 1.0 and *FindTar* version 2.0 (Table S4). *FindTar* version 1.0 uses the old criteria of seed homology plus free energy to predict MREs. FindTar version 2.0 uses new criteria, which adopts the central loop law as an additional criterion for MRE prediction. The MREs predicted by computational algorithms with or without the loop score were compared with the Gold Standard, the results of luciferase activity assays (Table 1). The sensitivity, specificity, and Youden Index were calculated (Table 1). Addition of the central loop scores increased specificity, decreased false positive rates, and reached Youden Index 1. However, it increased false negative rate slightly. Youden Index is a measure of the accuracy of a test based on its false positive and false negative rates, with the ideal value being 1.0. The results confirmed the effect of the loop scores in reducing the false positives and increasing the accuracy and specificity of MRE prediction.

**Table 1:** Statistical analyses of the results in MRE prediction before and after filtering with the central loop score

		number	old criteria 1		new criteria 2	
			+	−	+	−
**Gold Standard 3**	**+**	**45**	**44(TP)**	**1(FN)**	**39(TP)**	**6(FN)**
	−	**26**	**18(FP)**	**8(TN)**	**7(FP)**	**19(TN)**
Sensitivity (%)			97.8		86.7	
Specificity (%)			30.8		73.1 **	
Youden Index			0.286		0.597	

TP: true positive; FP: false positive; FN: false negative; TN: true negative.

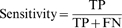
; 
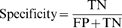

1. Old criteria: seed homology plus minimum free energy <−15 *kcal/mol,* not filtered by the central loop score. Detailed description can be found in the Methods section.

2. New criteria: seed homology plus free energy <−15 *kcal/mol,* filtered by the central loop score.

3. Gold Standard: Luciferase activity assay.

4. **: The specificity comparison between old criteria and new criteria shows a significant difference (p<0.01, statistics analysis using McNemar's Test).

Finally, we validated the importance of the loop score using published data[55], [56]. Lim LP and coworkers introduced chemically synthesized *miR-1*, *miR-124*, *miR-373*, *mir7*, *mir9*, *mir122a*, *mir128a*, *mir132*, *mir133a*, *mir142*, *mir148b*, and *mir181a* duplexes into HeLa cells, resulting in down-regulation of many mRNAs. The majority of the down-regulated mRNAs exhibit seed homologies with the introduced miRNAs. The experiment also showed that there are many mRNAs which have seeds homologies but are not under the regulation of the transfected miRNAs (false MREs). We used our revised *FindTar* algorithm (*FindTar* version 2.0) to calculate loop scores for the miRNA:MREs for all seed-containing mRNAs which were up- or down-regulated by the miRNAs used, and re-analyzed their microarray data (Table 2). Genes down-regulated or up-regulated at a p-value <0.05 at both 12hr and 24hr were scanned by FindTar for MREs of *miR-1*, *miR-124*, *miR-373*, *mir7*, *mir9*, *mir122a*, *mir128a*, *mir132*, *mir133a*, *mir142*, *mir148b*, or *mir181a*. The MRE criteria for *FindTar* version 1.0 includes seed homologies and free energy <−15, and *FindTar* version 2.0 includes seed homologies, free energy <−15, and loop score ≥20. The total number of bases in the 3′-UTR of the seed-containing mRNAs which were up- or down-regulated by the miRNAs used, were counted. The ratio of bases per MRE-Up/bases per MRE-D was used to evaluate the specificity of miRNA-mediated gene down regulation. Bases per MRE were calculated by the total number of bases in the 3′-UTR sequence of all affected mRNAs divided by the number of total MREs in these mRNAs. If all the mRNAs had seed homologies with the introduced miRNAs just by chance (the extreme case), bases per MRE-Up should be equal to bases per MRE-D. In this case, the ratio of bases per MRE-Up/bases per MRE-D would be close to 1, and the down-regulation of the messages would not have a relationship with the miRNAs. Otherwise, the ratio would be over 1 and the down-regulation of the messages would be considered at least partially mediated by the miRNAs used. This would be due to the presence of more MREs in the down-regulated mRNAs than in the up-regulated mRNAs, because of the specificity of the MREs. In this report, all the ratios are over 1.5, as shown in Table II. Furthermore, the ratios in group screened with the loop scores (L) are higher than those in unscreened group (NL). These results suggest that, besides seed sequence, loop scores also affect the specificity of miRNA-mediated gene regulation.

**Table 2:** Re-analysis of Lim *et al* and Grimson *et al.* microarray data from miRNAs transfected in HeLa cells.

miRNA	Gene Number (1)	Total Bases (2)	MRE(NL) (3)	MRE(L) (4)	Bases/MRE(NL) (5)	Bases/MRE (L) (6)	Ratio (7)
	D/U	D/U	D/U	D/U	D/U	D/U	NL/L*
miR-1	252/86	385947/152949	513 /107	267/49	752/1429	1445/3121	1.90/2.16
mir-124a	419/262	585637/459091	1891/909	787/330	309/505	744/1391	2.40/2.75
mir-373	234/197	343051/327288	889/591	392/237	385/553	875/1380	2.27/2.49
mir7	141/18	268092/26935	685/51	245/14	391/528	1094/1923	2.80/3.64
mir9	79/10	126313/10437	268/11	138/5	471/948	915/2087	1.94/2.20
mir122a	138/36	248250/44657	672/96	325/44	369/465	763/1014	2.07/2.18
mir128a	139/15	244848/28945	552/42	263/15	443/689	930/1929	2.10/2.80
mir132	215/26	365154/29761	550/28	239/10	663/1062	1527/2976	2.30/2.80
mir133a	456/264	772975/306917	1518/468	742/204	509/655	1041/1504	2.05/2.29

**Gene Number**: The number of genes down-regulated or up-regulated by *miR-1*, *miR-124a*, or *miR-373*, with p<0.05 at both 12 hr and 24 hr.

**D:** down-regulation. **U:** up-regulation.

**Total Bases:** The total number of bases from the 3′-UTR of the seed-containing mRNAs, which were either up- or down-regulated by *miR-1*, *miR-124a*, or *miR-373*.

**MRE (NL)** and **Bases/MRE(NL):** MRE (NL) indicates the MREs scanned by *FindTar* version-1 with an MRE criteria of seed homologies and free energy <−15, but without a loop score. Bases/MRE(NL) = Total Bases/Total MRE (NL).

**MRE (L)** and **Bases/MRE (L):** MRE (L) indicates MREs scanned by *FindTar* version-2 with an MRE criteria of seed homologies, free energy <−15, and loop score > = 20. Bases/MRE (L) = Total Bases/Total MRE (L).

**Ratio:** Ratio = Bases per MREup/Bases per MREdown. Ratio (NL) = (5)U/(5)D; Ratio (L) = (6)U/(6)D.

*: The ratio (NL) is significantly less than ratio (L) (p<0.05).

## Discussion

In the past few years, complementarity between miRNA and the 3′-UTR of target mRNAs, the conservation of target 3′-UTR sequences across different species, and the thermodynamics of the miRNA:MREs were widely used as the criteria to predict MREs in different computational algorithms, including miRanda [34], [37], TargetScan [31], DIANA-microT [32], and RNAhybrid [35]. The number of binding sites, the distance between sites, the cost of disruption to existing mRNA UTR secondary structure [57] and target-site accessibility [58] are also included as factors which might affect the accuracy of MRE prediction. However, the high levels of false positive prediction and the poor overlap of the predicted MREs between different computational algorithms were still common problems. We believed this was because some important rules governing miRNA:MRE interaction were not included.

Our results suggest that the location of central loop may be one of the important factors that have not been included. It is well known that binding of miRNAs with their targets is strongly affected by the nucleotides in the 5′ end of the miRNA:MRE duplex[53], [59], and that, in contrast to the strict requirement for base pairing at the proximal region, nucleotide mismatch at the distal region seems to be highly tolerated. However, there is hardly any previous discussion about the role of the loop region of the miRNA:MRE duplex on miRNA function, although Kiriakidou and coworkers have addressed the effect of the loop size on the efficiency of miRNA-mediated gene regulation[32]. In this investigation, we found that the location of the loop in the miRNA:mRNA duplexes is an important factor which affects the efficiency of gene regulation mediated by miRNAs. Loop scores combining both the size and location of the loops may be used as a new criterion for predicting MREs and their cognate miRNAs, resulting in a significant decrease in false positives.

The microarray data from Lim *et al.* and Grimson *et al.* provide strong support for the importance of the central loop rule[55], [56]. This can be shown through a mathematics approach. The number of predicted MREs in down-regulated genes is assigned to the T_D_ variable, with loop scores > = 20 as S_D_. The number of predicted MREs in up-regulated genes is then appointed as T_U,_ with loop scores > = 20 as S_U_. The ratio of positive MREs in down-regulated genes is named α, and the ratio in up-regulated genes β. Because miRNAs are down-regulative factors of gene expression, α should be bigger than β (α>β). Amongst the true positive MREs, the ratio of MREs with loop scores > = 20 is appointed m, and the false positive rate as n. According to the data in the Table 3, S_D_ = [αm+(1-α)n]T_D_, S_U_ = [βm+(1-β)n]T_U._ The total number of bases in the 3′-UTR in down-regulated genes is then assigned as B_D _and the total number of bases in up-regulated genes as B_U. _The ratio of bases per MRE-Up/bases per MRE-D in the group screened for their loop scores (loop score> = 20) is bigger than that in a non-screened group (NL), so:
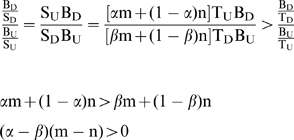
α>β, therefore m>n, meaning the ratio of MREs with loop scores > = 20 amongst true MREs is bigger than that in false MREs, so using the loop score as a criterion for MRE prediction is reasonable.

**Table 3:** Prediction of true and false MER with *FindTar*

	Down-regulated Genes		Up-regulated Genes	
	True Positive MER	False Positive MER	True Positive MER	False Positive MER
FindTar ver 1.0	αT_D_	(1-α)T_D_	βT_U_	(1-β)T_U_
FindTar ver 2.0	αmT_D_	(1-α)nT_D_	βmT_U_	(1-β)nT_U_

Because miRNA:mRNA duplexes with type I decentered loops usually have a slightly shorter seed region with 6–7 nucleotides, it may be argued that the decreased repressive efficiency of miRNAs with type I decentered loops is the result of their short seed regions causing a decreased binding affinity, rather than the location of the central loop. To address this issue, we compared the repressive effects of two groups of miRNAs on gene expression in Table 4. The miRNAs in both groups have a short seed region of 6–7 nucleotides, with group 1 having standard loops and group 2 with type I decentered loops. The results demonstrate that the repressive efficiency of group 1 is much stronger than group 2. These results suggest that loop location indeed affect the efficiency of miRNA regulation.

**Table 4:** Comparison of repressive ratio.

	microRNA/mRNA	IR%	Loop Score	Energy	Tail Score	Seed nts	Structure
Standard Loop	miR-15a/VEGF-Con2	34.05	20	−15.6	11	6	GUGUUUGGUAAUACACGACGAU:*||::*|||****|||||:**TTCAGGACAT----TGCTGTGC
	miR-16/VEGF-Con2	40.65	25	−22.7	10	6	GCGG--------UUAUAAAUGCACGACGAU *||:******************||||||**TGCTTTGGGGATTCCCTCCACATGCTGCAC
	miR-372/VEGF-Con2	50.18	20	−27.6	9	6	UGCGAGUUUACAGCG-------UCGUGAAA||||*||**|*||||*******:|||||**ACGCGCA--TCTCGCCCCCAGGGGCACTGC
	miR-140/VEGF-Con1	51.85	25	−15.7	10	7	GAUGGUAUCCCAUUUUGGUGA*************|||||||* ACGATCGATACAGAAACCACG
	miR-134/VEGF	45.06	25	−23.6	27	7	GGGAGACCAGUU--GGUCAGUGU *||*|||*||::***|||||||* TCC-CTGATCGGTGACAGTCACT
Type I Loop	miR-372-M1/VEGF-Con2	11.80	10	−27.5	27	6	TGCGAGTTTAGAGCG-------ACGTGATA||||****|||||||********|||||:* ACGC--GCATCTCGCCCCCAGGGGCACTGC
	miR-17-5p-M1/VEGF-Con1	-12.45	12.5	−29.5	37	7	UGAUG-GACGUGUCUACAGUGAAAC***||*||||:||||***|||||||GAGACTCTGCGCAGA--GCACTTTG
	miR-330/Cox2-Con4	9.03	0	−27.4	27	6	AGAGACGUCCGGCACAC--------GAAACG***|||||||||*||*|********:|||||GCACTGCAGGCC-TG-GTACTCAGATTTTGC
	miR-504/Cox2-Con4	16.58	10	−19	9	7	UAUCUCACGUCUGGUCCCAGA*******|||****|||||||TATATCAGCA--AAAGGGTCT
	miR-637/Cox2-Con4	15.86	10	−20.6	16	6	UGCGUCUCGGGCUUUCG---GGGGUCA**|||*||:::**|:|:***||:|||* TTGCA-AGTTTTCAGGTAAACCTCAGC
	miR-378/VEGF-Con2	−10.41	10	−29	27	7	UGUGUCCUGGACCU---CAGUCCUC:|||***:||||||****|||||||
p-value		0.0002	0.0001	0.1738	0.0794	0.7702	

* Statistics analysis by Student's test.

Evidence has shown that Ago, the major component of RISCs, includes both PAZ and PIWI domains. The PIWI domain of Ago, containing the triad of acidic amino acids DDH, is responsible for mRNA cleavage in RISCs, in which mRNA cleavage occurs between residues base-paired to nucleotides 10 and 11 of the siRNA, which is equivalent to the location of the standard loop of miRNA:mRNA duplexes. In contrast to the mRNA-cleaving RISC however, little is known about miRNPs or miRISCs. However, the position of the miRNA within the mature complex must be similar to that of the siRNA guide strand, because miRNPs can cleave mRNA when presented with highly complementary targets [60]. What factors decide whether mRNA will be cleaved or translationally repressed when bound by miRNP or an siRNA RISC is not clear, but we believe that the loop of the miRNA:mRNA duplex may be one of the important factors. One element is the location of the standard loop. It is known that perfect complementarity in the central part of the siRNA:mRNA duplexes allows A-form helix formation in the region facing the DDH triad, which is mandatory for cleavage [60]. The presence of a loop in this region effectively interferes with A-helix formation and would probably prevent cleavage. The other reason for the importance of a central loop in switching miRNPs or miRISCs from cleavage of the target to translational repression, is that the loop of the miRNA:mRNA duplex might affect the conformation of Ago. When siRNA or miRNA interact with Ago, its 5′ end binds to PIWI and the 3′ end to the PAZ domain of Ago. If there is flexible loop between the two ends, it would be easy for Ago to change its conformation to a new one which is necessary for translational repression.

Recently released data demonstrates that there are very complex interactions between miRNAs, mRNAs, miRNP proteins, and polyribosomes. It is reported that miRNP proteins, including Ago, are physically associated with the polyribosome, and that most miRNAs are also associated with polysomes. Association of miRNAs with polysomes is mRNA-mediated, with miRNAs associated with translating mRNAs, and a specific miRNA-targeted mRNA present in polysomes [61]–[63]. An miRNA:mRNA duplex with a flexible loop would have the ability to form different conformations to meet all of the needs in the complex process of translational repression.

## Materials and Methods

### MicroRNA and mRNA 3-′UTR Database

Human microRNA sequences (version 8.2) were retrieved from the miRBase website[64] (http://microrna.sanger.ac.uk/sequences/index.shtml). Sequences of the 3′-untranslated region (3′-UTR) of VEGF were retrieved from human (*Homo sapiens*), mouse (*Mus musculus*), rat (*Rattus norvegicus*), chimpanzee (*Pan troglodytes*), and Cow (*Bos taurus*) using the Ensembl Database (http://www.ensembl.org).

### Algorithm of FindTar Version1.0

We defined a relatively relaxed criterion for the seed region of the MRE so that more possible miRNA targets could be included in the primary screening. A flexible miRNA “seed window” (nt1–6, 2–7 and 3–8 counted from the 5′ end of the miRNA) was used to scan the 3′-UTRs of a gene in the order of 5′-3′ for potential target sites. In the seed windows, we searched for a perfectly Watson-Crick–base-paired stretch of 6 nt but tolerated one G-U wobble.

After the seed regions were identified, the upstream flanking region of the seed and the seed region itself was extracted for Dynamic programming. The miRNA:mRNA duplex and the MRE sequences of the VEGF 3′-UTR were identified with Dynamic hybridization. The distal sequences in the miRNA:mRNA duplex were scored with revised Gotoh's and Marks' methods [37], [65]. A score of +5 was given to G:C and A:T pairs, +2 to G:U wobble pairs, and −3 to mismatch pairs, with the gap-open and gap elongation parameters set to −8.0 and −2.0 respectively. The final scores of the miRNA:mRNA duplexes were the sum of single residue-pair match scores [37]. Then the sequences of MREs identified by Dynamic hybridization in the VEGF 3′-UTR and potential miRNA were used as input in RNAcofold to predict the RNA secondary structures of the miRNA:mRNA duplexes. If the length of the MRE identified by Dynamic programming was not long enough to form the miRNA:mRNA duplexes calculated by RNAcofold, the duplex would be simulated again using the same mRNA sequence. This time, the sequence used would begin further upstream, such that the region used was 40nt in length including the seed region.

RNAcofold most lately released in the Vienna RNA Package 1.5 beta version (www.tbi.univie.ac.at/ivo/RNA/) was incorporated into the *FindTar* version 1.0 algorithm. Sequences of MREs in the VEGF 3′-UTR and potential miRNA were input to precisely predict the RNA secondary structures of the miRNA:mRNA duplexes by calculating the minimum free energy (ΔG) of the whole miRNA:mRNA duplex.

### Algorithm of FindTar Version2.0

In order to decrease the number of false positives in the prediction of putative miRNA targets, the central loop law was introduced into the *FindTar* version 1.0 to produce *FindTar Version2.0* (http://bio.sz.tsinghua.edu.cn/findtar/). There were three elements in the central loop law: loop position, loop size, and loop priority. (1) Loop position coefficient: 0 for loops starting before nt7 inclusive, 0.5 for loops starting at nt8, both named Type I decentered loops; 1 for loops starting between nt9 and nt11 inclusive, named the standard loop; 0.75 for loops starting after nt12 inclusive, named the Type II decentered loop. (2) Loop size coefficient: 10 for loops with a size of 1 bp, 20 for 2 bp, 25 for 3 bp. When the loop size is above 4 bp inclusive, the coefficient is back to 20. (3) Loop priority law: this criterion was defined to avoid the “noise” bulge which may confuse the central loop scoring system. In order to make it clear which loop should be scored and which should not, the priority for scoring a loop was defined as the first loop which met the following criteria: (*a*) the first loop starting before nt9 of more than 1 bp; (*b*) the first loop starting between nt9 and nt11 of any size–if there is more than one loop, then the longest loop will be chosen; (*c*) the first loop starting after nt11 of any size. Finally, the total loop score was calculated by multiplying the location coefficient with the size coefficient. The central loop score was applied as a filter after all the miRNA:MREs were predicted. All these duplexes were scored, with a higher score equivalent to a higher probability of functioning.

### Preparation of Reporter Vectors

The construction of luciferase report vectors, named pRL-VEGF-Con1 and pRL-VEGF-Con2, has been described previously[40]. Briefly, two fragments of the VEGF 3′-UTR with multiple MREs were PCR-amplified using primers with the following sequences: construct 1 sense, 5′-cgttctagagtttcgggaaccagatctc; antisense, 5′-aacactagtaatgcttccgccggagt; construct 2 sense, 5′-tcttctagacaggtcagacggacag; antisense, 5′-acaactagtctcttctcttcgccgg. Two copies of each fragment were cloned downstream of the stop codon in pRL-TK (Promega Corp., Madison, WI, USA). pRL-VEGF-Con1 contains nucleotides nt31-216 of the VEGF 3′-UTR, while pRL-VEGF-Con2 contains nucleotides nt703-944 of the VEGF 3′-UTR. The site-specific mutants of pRL-VEGF-Con1 and pRL-VEGF-Con2 were also established by using Takara MutanBEST Kit (Takara Corp., Dalian, PRC) according the method provided by the manual.

### Cell Culture and Hypoxia Induction

COS-7 cells were cultured in Dulbecco's modified Eagle medium (DMEM) (GIBCO, Carlsbad, CA, USA) containing 10% fetal bovine serum (GIBCO, Carlsbad, CA, USA), and the CNE cells obtained from Kunming Cell Bank (Kunming, China) were cultured in RPMI 1640 (GIBCO, Carlsbad, CA, USA) containing 10% fetal bovine serum. Hypoxia induction was performed by adding desferrioxamine mesylate (DFOM) (Sigma-Aldrich Co., Saint Louis, MO, USA) in the culture medium to a final concentration of 130 µM.

### Preparation of an siRNA duplex homologous to the miRNA sequence, transfection and dual luciferase assay

SiRNA duplex homologous to the miRNA sequences were synthesized and purified by Shanghai GenePharma Co. Ltd (Shanghai, China). pRL-TK plasmids bearing MREs in the 3′-UTR (400 ng for CNE, 100 ng for COS-7) were co-transfected with the pGL-3 reporter plasmid (100 ng for CNE, 5 ng for COS-7) and 20 pmol of siRNA/miRNAs into CNE or COS-7 cells (∼3×10^4^, 24-well cell culture plates) using Lipofectamine 2000 (Invitrogen, Carlsbad, CA) in accordance with the manufacturer's instructions. 26–32 hours after transfection, luciferase activity was determined using the Dual Luciferase Reporter Assay System (Promega Corp., Madison, WI, USA).

### ELISA assay

The supernatant harvested from cell culture 30 hrs after transfection was assayed using human VEGF ELISA kits from R&D Systems (Minneapolis, MN, USA) and read by GENios ELISA Microplate Reader (TECAN, Austria). All experiments were repeated in triplicate.

## Supporting Information

Table S1 (0.07 MB DOC)

Table S2 (0.09 MB DOC)

Table S3 (0.07 MB DOC)

Table S4 (0.14 MB DOC)
