# Supplementary material for: The Effect of Central Loops in miRNA:MRE Duplexes on the Efficiency of miRNA-Mediated Gene Regulation
Source: PLoS One. 2008 Mar 5;3(3):e1719. doi: 10.1371/journal.pone.0001719 (PMC2248708; doi:10.1371/journal.pone.0001719)
Supplement: Table S1 — (0.07 MB DOC) [file pone.0001719.s001.doc]

Table S1. MiRNAs in group 1, 2 and their putative MREs in VEGF

| 1. group1 |  |  | |  | |  | |  | |  |
| --- | --- | --- | --- | --- | --- | --- | --- | --- | --- | --- |
| ***microRNA*** | Δ**G *(kcal/mol)*** | ***MRE Position*** | | ***Loop type*** | | ***Tail Score*** | | ***Structure*** | |  |
| hsa-miR-16 | -25.6 | 259-283 | | S | | 28 | | GCGGU--UAUAAA-UGCACGACGAU  |||||***|||||**|*||||||||  CGCCATTTTATTTTTCTTGCTGCTA | |  |
| hsa-miR-17-5p | -27.5 | 165-184 | | S | | 29 | | UGAUG-GACGUGACAUUCGUGAAAC  ***||*||||:|***:|||||||||  GAGACTCTGCGC—AGAGCACTTTG | |  |
| hsa-miR-20a | -25.9 | 166-184 | | S | | 23 | | GAUG-GACGUGAUAUUCGUGAAAU  **||*||||:|***:||||||||:  AGACTCTGCGC—AGAGCACTTTG | |  |
| hsa-miR-20b | -29.3 | 166-184 | | S | | 26 | | GAUG-GACGUGAUACUCGUGAAAC  **||*||||:|***||||||||||  AGACTCTGCGC—AGAGCACTTTG | |  |
| hsa-miR-34a | -27.2 | 831-850 | | II | | 15 | | UUGUUGGUCGAUUCUGUGACGGU  *****||||***:|:|||||||*  CGCCCCCAG---GGGCACTGCCT | |  |
| hsa-miR-34b | -24.5 | 833-851 | | II | | 16 | | GUUAGUCGAUUACUGUGACGGAU  ****|||*****|:||||||||:  CCCCCAG----GGGCACTGCCTG | |  |
| hsa-miR-93 | -24.1 | 163-183 | | S | | 19 | | GAUG-GACGUGCUUGUCGUGAAA  **||*||||:|**|*||||||||  AGACTCTGCGC—AGAGCACTTT | |  |
| hsa-miR-106a | -25.9 | 162-184 | | S | | 25 | | CGAUG-GACGUGACAUUCGUGAAAA  ***||*||||:|***:||||||||*  GAGACTCTGCGC—AGAGCACTTTG | |  |
| hsa-miR-106b | -24.6 | 166-184 | | S | | 26 | | UAGACGUGACAGUCGUGAAAU  *|||||:|****||||||||:  CTCTGCGC—AGAGCACTTTG | |  |
| hsa-miR-125a | -28.6 | 5-32 | | S | | 17 | | GUGUCCAAUUUCC-C----AGAGUCCCU  *:||||**:||||*|*****|||||||*  GGCAGGAGGAAGGAGCCTCCCTCAGGGT | |  |
| hsa-miR-150 | -25.5 | 484-506 | | S | | 24 | | GUGAC-CAUGUUCCCAACCCUCU  *:|||*|*||**|*||||||||*  TGCTGTGGACTTGAGTTGGGAGG | |  |
| hsa-miR-195 | -24.5 | 260-283 | | S | | 30 | | CGGU--UAUAAAGA-CACGACGAU  ||||***|||||:|**||||||||  GCCATTTTATTTTTCTTGCTGCTA | |  |
| hsa-miR-330 | -24.0 | 774-800 | | S | | 20 | | AGAGACGUCC---GGC-ACACGAAACG  ***||*||||****:|*|||||||||*  GAGCTTCAGGACATTGCTGTGCTTTGG | |  |
| hsa-miR-378 | -29.0 | 842-864 | | I | | 27 | | UGUGUCCUGGACCU---CAGUCCUC  :|||***:||||||****|||||||  GCAC—TGCCTGGAAGATTCAGGAG | |  |
| hsa-miR-520g | -25.0 | 163-185 | | II | | 29 | | UGUGAGAUUUCCCUUCGUGAAACA  **|||||:*****:|||||||||*  AGACTCTG-CGCAGAGCACTTTGG | |  |
| 1. group 2 |  | |  | |  | |  | |  | |
| ***microRNA*** | ***Energy (kcal/mol)*** | | ***MRE Position*** | | ***Loop form*** | | ***Tail Score*** | | ***Structure*** | |
| hsa-miR-15a | -19.5 | | 255-283 | | S | | 11 | | GUGUUUGGUAA-------UACACGACGAU  *|:**:|||||**********||||||||  GATTCGCCATTTTATTTTTCTTGCTGCTA | |
| hsa-miR-15b | -17.3 | | 770-795 | | S | | 10 | | ACAUUUGG--UACU-ACACGACGAU  *|**|:|:****||***|||||:**  GGGGAGCTTCAGGACATTGCTGTGC | |
| hsa-miR-29b | -19.4 | | 1729-1749 | | S | | 28 | | UUGUGACUAAAGUUUACCACGAU  **||:|*||||****||||||||  ATCATTTATTT—ATTGGTGCTA | |
| hsa-miR-107 | -22.5 | | 771-794 | | II | | 29 | | ACUAUCGGG-ACAUGUUACGACGA  *|**|||::****|||*|||||:*  GGGGAGCTTCAGGACATTGCTGTG | |
| hsa-miR-134 | -23.6 | | 1414-1435 | | S | | 27 | | GGGAGACCAGUU--GGUCAGUGU  *||*|||*||::***|||||||*  TCC-CTGATCGGTGACAGTCACT | |
| hsa-miR-140 | -20.2 | | 21-45 | | S | | 24 | | GAUGGUAUCCCA----UUUUGGUGA  ||*||**|||||****::|||||**  CTCCCTCAGGGTTTCGGGAACCAGA | |
| hsa-miR-147 | -20.3 | | 794-817 | | S | | 11 | | CGUCUUCG----------UAAAGGUGUGUG  ||****||*************||||||::|  GC-TGTGCTTTGGGGATTCCCTCCACATGC | |
| hsa-miR-205 | -23.7 | | 521-545 | | I | | 28 | | GUCUGAGGC--CACCUU-ACUUCCU  ||||**|:|****||||**|:||||  CAGATCCTGACAGGGAAGAGGAGGA | |
| hsa-miR-302d | -22.8 | | 198-218 | | II | | 13 | | UGUGAGUUUGUACCUUCGUGAAU  **||||**:|**|||||||:|**  AGACTCCGGC—GGAAGCATTCC | |
| hsa-miR-361 | -20.4 | | 1605-1625 | | II | | 27 | | CAUGGGGACCUCUAAGACUAUU  |||:***||**||||||||||*  GTATATATG-TGATTCTGATAA | |
| hsa-miR-372 | -19.7 | | 163-183 | | S | | 9 | | UGC-GAGUUUACAGCG--UCGUGAAA  **|*|||***||*|||**||||||||  GAGACTC---TG-CGCAGAGCACTTT | |
| hsa-miR-373 | -25.7 | | 823-848 | | S | | 20 | | UGUGGGGUUUUAGCUUCGUGAAG  *|:||||******|::|||||**  TCGCCCC----CAGGGGCACTGC | |
| hsa-miR-383 | -18.3 | | 1397-1423 | | S | | 17 | | UCGG-UGUUAG----UGGAAGACUAGA  *|::**|||||*****|:**||||||*  TGTTTCCAATCTCTCTCTCCCTGATCG | |
| hsa-miR-504 | -18.7 | | 1272-1292 | | S | | 12 | | UAUCUCACGUCUGGUCCCAGA  *********|||*:|||||:|  TTTAATTAAAGAGTAGGGTTT | |
| hsa-miR-520h | -23.9 | | 165-185 | | II | | 27 | | UGAGAUUUCCCUUCGUGAAACA  |||||:*****:|||||||||*  ACTCTG-CGCAGAGCACTTTGG | |

Note: S: standard central loop; I: type I decentered loop; II: type II decentered loop.
