# Supplementary material for: The Effect of Central Loops in miRNA:MRE Duplexes on the Efficiency of miRNA-Mediated Gene Regulation
Source: PLoS One. 2008 Mar 5;3(3):e1719. doi: 10.1371/journal.pone.0001719 (PMC2248708; doi:10.1371/journal.pone.0001719)
Supplement: Table S2 — (0.09 MB DOC) [file pone.0001719.s002.doc]

Table S2. Recalculation of proved targets

| ***No.*** | ***miRNA*** | ***Gene*** | ***MRE Position*** | ***Loop type*** | ***Free energy*** | ***Predictive miRNA:MRE Structure*** | ***Reference*** |
| --- | --- | --- | --- | --- | --- | --- | --- |
| **1** | let-7b | lin-28 | 886-912 | S | -30.6 | UUGGUGUGUUGGA-----UGAUGGAGU  :|*::||||:|||*****|||||||||  GAGTGCACAGCCTATTGAACTACCTCA | [1] |
| **2** | miR-141 | Clock | 216-233 | S | -18.7 | GGUAGAAAUGGUCUGUCACAAU  |||***||*|:*|||||||||*  CCA--GTT-CT-GACAGTGTTT | [1] |
| **3** | miR-24 | MAPK14 | 645-669 | S | -30.7 | GACAAGGACGACUU---GACUCGGU  |||***||||||||***|||||||*  CTGCAGCTGCTGAAGTTCTGAGCCG | [1] |
| **4** | miR-145 | FLJ21308 | 29-56 | S | -25.8 | UUCCCUAAGGA-CCC---UUUUGACCUG  ******|||||*|||***|||:|||||:  AAAGCCTTCCTCGGGTTCAAAGCTGGAT | [1] |
| **5** | let-7e | SMC1L1 | 68-91 | S | -25.2 | UGAUAUGUUGGA----GGAUGGAGU  |||***||||||****||||||||*  ACT-CCCAACCTTCCCCCTACCTCC | [1] |
| **6** | miR-15 | DMTF1 | 126-145 | S | -24.1 | GUGUUUGGUAAUACACGACGAU  ||||***||||:|*||||||||  CACA-CACATTGT-TGCTGCTA | [1] |
| **7^#^** | miR-15 | CGI-38 | 288-308 | I | -23.8 | GCGG-UUAUAAAUGCACGACGAU  *|||**|||||||***||||||:  TGCCTCATATTTA--AGCTGCTG | [1] |
| **8^a^** | miR-15 | CGI-38 | 288-311 | S | -24.9 | GCGG-UUAUAAAU-GCACGACGAU  *|||**|||||||***|||||||*  TGCCTCATATTTAAGCTGCTGCTC | [1] |
| **9** | miR-143 | ERK5 | 108-127 | S | -23.3 | ACUCGAUGUCACGAAGUAGAGU  ****||:|||*|*||||||||*  TATTCTGCAG-G-TTCATCTCA | [2] |
| **10** | miR-1 | hand2 | 204-232 | S | -14.5 | AUGUAUGAAGAAAUGUAAGGU  *************:||||||*  TATTTGAAGAAAAGCATTCCA | [3] |
| **11** | miR-1 | TMSB4X | 17-36 | S | -20.6 | AUGUAUGAAGAAAUGUAAGGU  *|:||:|**||*||||||||*  AATATGC-ACTGTACATTCCA | [3] |
| **12** | miR-15 | BCL2 | 2515-2536 | S | -23.0 | GUGUUUGGUAAUACACGACGAU  ************||||||||||  AATATCCAATCCTGTGCTGCTA | [4] |
| **13** | miR-16 | BCL2 | 2517-2536 | S | -22.4 | GCGGUUAUAAAUGCACGACGAU  **|||||*****:|||||||||  ATCCAAT---CCTGTGCTGCTA | [4] |
| **14** | miR-10a | HOXA1 | 954-976 | S | -17.6 | GUGUUUAAGCCUAGAUGUCCCAU  ***************|||||||:  GTTTAGGTTACTAAAACAGGGTG | [5] |
| **15** | miR-155 | AGTR1 | 70-90 | S | -15.5 | GGGGAUAGUGCUAAUCGUAAUU  ***|||*||*****|||||||*  TCACTACCA-AATGAGCATTAG | [6] |
| **16^b^** | miR-375 | C1qbp | 32-54 | S | -19.1 | AGUGCG--CUCGGCUUGCUUGUUU  *||*||***:|||*|::||||||*  GCAAGCUUUGGCC-AGUGAACAAA | [7] |
| **17^b^** | miR-375 | C1qbp | 209-225 | S | -15.1 | AGUGCGCUCGGCUUGCUUGUUU  *||:|*****:|*|:|:||||*  ACAUG----AUG-AUGGACAAU | [7] |
| **18^b^** | miR-375 | Usp1 | 197-217 | I | -20.4 | AGUGC-GCUCGGCUUGCUUGUUU  ****|*:||||:|:**||||||*  GAUGGAUGAGCUGG--GAACAAA | [7] |
| **19** | miR-221 | KIT | 1010-1037 | S | -21.8 | CUUUGGGUCG--UCUG---UUACAUCGA  ****|::||:**:||:****|||||||*  CCATCTTAGTTTGGATTCTTATGTAGCA | [8] |
| **20** | miR-222 | KIT | 997-1037 | S | -22.7 | CUCU-GGGUCAUCGG----------------UCUACAUCGA  |:||***||***|||******************|||||||*  GGGAAAACA-CTGCCATCTTAGTTTGGATTCTTATGTAGCA | [8] |

Note:

a: two different structural forms in one miRNA target location; b: *Mus musculus* microRNA.

S: standard central loop; I: type I decentered loop; II: type II decentered loop.

References:

1. Kiriakidou, M., et al., *A combined computational-experimental approach predicts human microRNA targets.* Genes Dev, 2004. **18**(10): p. 1165-78.

2. Esau, C., et al., *MicroRNA-143 regulates adipocyte differentiation.* J Biol Chem, 2004. **279**(50): p. 52361-5.

3. Zhao, Y., E. Samal, and D. Srivastava, *Serum response factor regulates a muscle-specific microRNA that targets Hand2 during cardiogenesis.* Nature, 2005. **436**(7048): p. 214-20.

4. Cimmino, A., et al., *miR-15 and miR-16 induce apoptosis by targeting BCL2.* Proc Natl Acad Sci U S A, 2005. **102**(39): p. 13944-9.

5. Garzon, R., et al., *MicroRNA fingerprints during human megakaryocytopoiesis.* Proc Natl Acad Sci U S A, 2006. **103**(13): p. 5078-83.

6. Martin, M.M., et al., *Microrna-155 regulates human angiotensin II type 1 receptor expression in fibroblasts.* J Biol Chem, 2006.

7. Krek, A., et al., *Combinatorial microRNA target predictions.* Nat Genet, 2005. **37**(5): p. 495-500.

8. Voorhoeve, P.M., et al., *A genetic screen implicates miRNA-372 and miRNA-373 as oncogenes in testicular germ cell tumors.* Cell, 2006. **124**(6): p. 1169-81.
