# Supplementary material for: The Effect of Central Loops in miRNA:MRE Duplexes on the Efficiency of miRNA-Mediated Gene Regulation
Source: PLoS One. 2008 Mar 5;3(3):e1719. doi: 10.1371/journal.pone.0001719 (PMC2248708; doi:10.1371/journal.pone.0001719)
Supplement: Table S3 — (0.07 MB DOC) [file pone.0001719.s003.doc]

Table S3. MREs in pRL-VEGF-Con1 and pRL-VEGF-Con2

| 1. pRL-VEGF-Con1 | |  | | |  |  | |  | |
| --- | --- | --- | --- | --- | --- | --- | --- | --- | --- |
| ***miRNA*** | | ***IR%*** | | | ***MRE Location*** | ***Central Loop Score*** | | ***Structure*** | |
| **hsa-miR-17-5p** | | 39.07 | | | 165-184 | 25 | | UGAUG-GACGUGACAUUCGUGAAAC  ***||*||||:|***:|||||||||  GAGACTCTGCGC--AGAGCACTTTG | |
| **hsa-miR-20a** | | 38.90 | | | 166-184 | 25 | | GAUG-GACGUGAUAUUCGUGAAAU  **||*||||:|***:||||||||:  AGACTCTGCGC--AGAGCACTTTG | |
| **hsa-miR-20b** | | 41.84 | | | 166-184 | 25 | | GAUG-GACGUGAUACUCGUGAAAC  **||*||||:|***||||||||||  AGACTCTGCGC--AGAGCACTTTG | |
| **hsa-miR-34a** | | 19.68 | | | 72-99 | 20 | | UUGUUG-GUCGAUUCUGUGACGGU **|:|:*|||*****||:|||||*  ATCGATACAGAAACCACGCTGCCG | |
| **hsa-miR-34b** | | 28.61 | | | 78-100 | 20 | | GUUA-GUCGAUUACUGUGACGGAU |:||*|||******||:|||||**  CGATACAG-AAACCACGCTGCCGC | |
| **hsa-miR-93** | | 56.03 | | | 163-183 | 25 | | GAUG-GACGUGCUUGUCGUGAAA  **||*||||:|**|*||||||||  AGACTCTGCGC--AGAGCACTTT | |
| **hsa-miR-106a** | | 46.00 | | | 162-184 | 25 | | CGAUG-GACGUGACAUUCGUGAAAA  ***||*||||:|***:||||||||*  GAGACTCTGCGC--AGAGCACTTTG | |
| **hsa-miR-106b** | | 34.38 | | | 166-184 | 20 | | UAGACGUGACAGUCGUGAAAU  *|||||:|****||||||||:  CTCTGCGC--AGAGCACTTTG | |
| **hsa-miR-140** | | 51.85 | | | 74-93 | 25 | | GAUGGUAUCCCAUUUUGGUGA  *************|||||||*  ACGATCGATACAGAAACCACG | |
| **hsa-miR-205** | | 23.47 | | | 138-157 | 10 | | GUCUGAGGCCACCUUACUUCCU  ||||**||**||*|||||||||  CAGAAACC--TGAAATGAAGGA | |
| **hsa-miR-302d** | | 54.71 | | | 163-183 | 25 | | UGUGAGUUUGUACCUUCGUGAAU  **||||**:|:***:|||||||*  AGACTC-TGCG-CAGAGCACTTT | |
| **hsa-miR-372** | | 30.84 | | | 163-183 | 20 | | UGC-GAGUUUACAGCG--UCGUGAAA  **|*|||***||*|||**||||||||  GAGACTC---TG-CGCAGAGCACTTT | |
| **hsa-miR-373** | | 41.76 | | | 163-183 | 20 | | UGUGGGGUUUUAGCUUCGUGAAG  **||:|::****|**|||||||:  AGACTCTG--CGCAGAGCACTTT | |
| **hsa-miR-520g** | | 20.80 | | | 163-185 | 15 | | UGUGAGAUUUCCCUUCGUGAAACA  **|||||:*****:|||||||||*  AGACTCTG-CGCAGAGCACTTTGG | |
| **hsa-miR-520h** | | 23.53 | | | 165-185 | 15 | | UGAGAUUUCCCUUCGUGAAACA  |||||:*****:|||||||||*  ACTCTG-CGCAGAGCACTTTGG | |
| 1. pRL-VEGF-Con2 | | |  |  | | |  | |  |
| ***miRNA*** | ***IR%*** | | | ***MER Location*** | | | ***Central Loop Score*** | | ***Structure*** |
| **hsa-miR-15a** | 34.06 | | | 778-795 | | | 20 | | GUGUUUGGUAAUACACGACGAU  :*||::*|||****|||||:**  TTCAGGACAT----TGCTGTGC |
| **hsa-miR-16** | 40.65 | | | 793-822 | | | 25 | | GCGG--------UUAUAAAUGCACGACGAU  *||:******************||||||**  TGCTTTGGGGATTCCCTCCACATGCTGCAC |
| **hsa-miR-17-5p** | 57.23 | | | 776-799 | | | 25 | | UGAUGGAC-GUGACAUUCGUGAAAC  :||*|**|*||:||***|::|||||  GCTTCAGGACATTG-CTGTGCTTTG |
| **hsa-miR-20b** | 44.31 | | | 777-799 | | | 25 | | GAUGGAC-GUGAUACUCGUGAAAC  ******|*||:|:***|::|||||  CTTCAGGACATTG-CTGTGCTTTG |
| **hsa-miR-140** | 38.99 | | | 902-918 | | | 25 | | GAUGGUAUCCCAUUUUGGUGA  *|:||**|||***|::|||||  TTGCC-CAGG---AGGCCACT |
| **hsa-miR-147** | 49.90 | | | 794-817 | | | 25 | | CGUCUUCG----------UAAAGGUGUGUG  ||****||*************||||||::|  GC-TGTGCTTTGGGGATTCCCTCCACATGC |
| **hsa-miR-205** | 17.75 | | | 724-749 | | | 18.75 | | GUCUGAGG-CCA---CCUUACUUCCU  ||||:**|*|||***||:||||:|**  CAGATCACAGGTACAGGGATGAGGAC |
| **hsa-miR-330** | 17.54 | | | 768-800 | | | 10 | | AGAGACGUCC---GGC-ACACGAAACG  ***||*||||****:|*|||||||||*  GAGCTTCAGGACATTGCTGTGCTTTGG |
| **hsa-miR-372** | 50.18 | | | 821-848 | | | 20 | | UGCGAGUUUACAGCG-------UCGUGAAA  ||||*||**|*||||*******:|||||**  ACGCGCA--TCTCGCCCCCAGGGGCACTGC |
| **hsa-miR-373** | 39.32 | | | 825-848 | | | 20 | | UGUGGGGUUUUAGCUUCGUGAAG  *|:||||******|::|||||**  TCGCCCC----CAGGGGCACTGC |
| **hsa-miR-378** | -10.41 | | | 842-864 | | | 10 | | UGUGUCCUGGACCU---CAGUCCUC  :|||***:||||||****|||||||  GCAC--TGCCTGGAAGATTCAGGAG |
| **hsa-miR-520g** | 45.78 | | | 774-800 | | | 20 | | UGUGAGAUUUCC-----CUUCGUGAAACA  **:||:***|||*****|**|::|||||*  GAGCTT--CAGGACATTGCTGTGCTTTGG |
| **hsa-miR-520h** | 31.62 | | | 776-800 | | | 20 | | UGAGAUUUCC-----CUUCGUGAAACA  :||:***|||*****|**|**|||||*  GCTT--CAGGACATTGCTGTGCTTTGG |
