# Supplementary material for: The Effect of Central Loops in miRNA:MRE Duplexes on the Efficiency of miRNA-Mediated Gene Regulation
Source: PLoS One. 2008 Mar 5;3(3):e1719. doi: 10.1371/journal.pone.0001719 (PMC2248708; doi:10.1371/journal.pone.0001719)
Supplement: Table S4 — (0.14 MB DOC) [file pone.0001719.s004.doc]

Table S4. Data analysis of all single MREs

| **microRNA** | **Gene** | **FindTar version 1.0** | **FindTar version 2.0** | **Repressive Effect** |
| --- | --- | --- | --- | --- |
| **A** |  |  |  |  |
| *let-7a | LIN-41a | Y | Y | Y |
| let-7a | LIN-41b | Y | Y | Y |
| let-7b | LIN-28.wt | Y | Y | Y |
| *let-7b | LIN-28.M1 | N | N | N |
| *let-7b | LIN-28.M2 | N | N | N |
| *let-7b | LIN-28.M3 | Y | Y | Y |
| let-7b | LIN-28.M4 | Y | N | N |
| let-7b | LIN-28.M5 | Y | N | N |
| let-7b | LIN-28.M6 | Y | N | N |
| let-7b | LIN-28.M7 | Y | Y | Y |
| let-7b | LIN-28.M8 | Y | Y | Y |
| let-7b | LIN-28.M9 | Y | Y | Y |
| let-7b | LIN-28.M10 | Y | Y | Y |
| let-7b | LIN-28.M11 | Y | Y | Y |
| let-7b | LIN-28.M12 | Y | Y | Y |
| let-7b | LIN-28.M13 | Y | Y | Y |
| let-7b | LIN-28.M14 | Y | Y | Y |
| let-7b | LIN-28.M15 | Y | Y | N |
| let-7b | LIN-28.M16 | Y | Y | N |
| let-7b | LIN-28.M17 | Y | N | Y |
| let-7b | LIN-28.M18 | Y | Y | Y |
| let-7b | LIN-28.M19 | Y | Y | N |
| let-7b | LIN-28.M20 | Y | Y | N |
| let-7b | LIN-28.M21 | Y | Y | N |

| **B** | |  | | | |  | | | |  | |  | |  |
| --- | --- | --- | --- | --- | --- | --- | --- | --- | --- | --- | --- | --- | --- | --- |
| miR-141 | | CLOCK | | | | Y | | | | Y | | Y | |  |
| miR-24 | | MAPK14 | | | | Y | | | | Y | | Y | |  |
| miR-145 | | FLJ21308 | | | | Y | | | | Y | | Y | |  |
| miR-23a | | FLJ13158 | | | | Y | | | | Y | | Y | |  |
| let-7e | | SMC1L1 | | | | Y | | | | Y | | Y | |  |
| miR-15 | | hDMP1 | | | | Y | | | | N | | Y | |  |
| miR-16 | | CGI-38 | | | | Y | | | | Y | | Y | |  |
| miR-199b | | LAMC2 | | | | Y | | | | N | | Y | |  |
| *miR-103 | | FBXW1B | | | | N | | | | N | | Y | |  |
| **C**  hsa-miR-17-5pM1 | | | Con1 | | | | Y | | | | N | | N |  |
| hsa-miR-17-5pM2 | | | Con1 | | | | Y | | | | N | | N |  |
| hsa-miR-29b | | | Con1 | | | | N | | | | N | | N |  |
| hsa-miR-150 | | | Con1 | | | | N | | | | N | | N |  |
| hsa-miR-29b | | | Con2 | | | | N | | | | N | | N |  |
| hsa-miR-106a | | | Con2 | | | | N | | | | N | | N |  |
| hsa-miR-134 | | | Con2 | | | | N | | | | N | | N |  |
| hsa-miR-372M1 | | | Con2 | | | | Y | | | | N | | N |  |
| hsa-miR-372M2 | | | Con2 | | | | Y | | | | N | | Y |  |
| hsa-miR-383 | | | Con2 | | | | N | | | | N | | N |  |
| **D**  hsa-miR-17-5p | Con1 | | | | Y | | | | Y | | | Y | |  |
| hsa-miR-20a | Con1 | | | | Y | | | | Y | | | Y | |  |
| hsa-miR-20b | Con1 | | | | Y | | | | Y | | | Y | |  |
| hsa-miR-34a | Con1 | | | | Y | | | | Y | | | N | |  |
| hsa-miR-34b | Con1 | | | | Y | | | | Y | | | Y | |  |
| hsa-miR-93 | Con1 | | | | Y | | | | Y | | | Y | |  |
| hsa-miR-106a | Con1 | | | | Y | | | | Y | | | Y | |  |
| hsa-miR-106b | Con1 | | | | Y | | | | Y | | | Y | |  |
| hsa-miR-140 | Con1 | | | | Y | | | | Y | | | Y | |  |
| hsa-miR-205 | Con1 | | | | Y | | | | N | | | N | |  |
| hsa-miR-302d | Con1 | | | | Y | | | | Y | | | Y | |  |
| hsa-miR-372 | Con1 | | | | Y | | | | Y | | | Y | |  |
| hsa-miR-373 | Con1 | | | | Y | | | | Y | | | Y | |  |
| hsa-miR-520g | Con1 | | | | Y | | | | N | | | Y | |  |
| hsa-miR-520h | Con1 | | | | Y | | | | N | | | N | |  |
| **E**  hsa-miR-15a | Con2 | | | Y | | | | Y | | | | Y | | |
| hsa-miR-16 | Con2 | | | Y | | | | Y | | | | Y | | |
| hsa-miR-17-5p | Con2 | | | Y | | | | Y | | | | Y | | |
| hsa-miR-20b | Con2 | | | Y | | | | Y | | | | Y | | |
| hsa-miR-140 | Con2 | | | Y | | | | Y | | | | Y | | |
| hsa-miR-147 | Con2 | | | Y | | | | Y | | | | Y | | |
| hsa-miR-205 | Con2 | | | Y | | | | N | | | | N | | |
| hsa-miR-330 | Con2 | | | Y | | | | N | | | | N | | |
| hsa-miR-372 | Con2 | | | Y | | | | Y | | | | Y | | |
| hsa-miR-373 | Con2 | | | Y | | | | Y | | | | Y | | |
| hsa-miR-378 | Con2 | | | Y | | | | N | | | | N | | |
| hsa-miR-520g | Con2 | | | Y | | | | Y | | | | N | | |
| hsa-miR-520h | Con2 | | | Y | | | | Y | | | | Y | | |

**Con1**: pRL-VEGF-Con1; **Con2**: pRL-VEGF-Con2

**Y** in FindTar-1/2: predicted as MRE; **N** in FindTar-1/2: unpredicted as MRE.

1. Re-analysis of the data from the Figure 1 of Kiriakidou’s paper.
2. Re-analysis of the data from the Figure 3 and 6 of Kiriakidou’s paper
3. Data analysis of other miRNA:MREs
4. Data analysis of Figure 2A
5. Data analysis of Figure 2B

Notes:

1. Table S4 A data originates from Fig. 1 of *a combined computational-experimental approach predicting human microRNA targets*, Kiriakidou *et al.* 2004.
2. Table S4B data originates from Fig. 3 and Fig. 6A of *a combined computational-experimental approach predicting human microRNA targets*, Kiriakidou *et al.* 2004.
3. The miRNA with asterixes (*) indicates that we cannot predict the target by using *FindTar*. *FindTar* predicts miRNA targets and needs a consecutive 6mer perfect match in the seed region. Any target not conforming to this criterion will not be found.
4. Repressive Effect: The results of the luciferase activity assay which were used as the gold standard in the statistical analysis.
